# Reporting on the establishment of a Privacy Preserving Record Linkage to Facilitate an Ongoing Crosswalk Between Research and Health Administrative Databases

**DOI:** 10.23889/ijpds.v9i5.2760

**Published:** 2024-09-18

**Authors:** Brendan Behan, Alana Sparks, Heena Cheema, Sibel Naska, Francis Jeanson, Shalane Basque, Charlotte Ma, Jason Chai-Onn, Mojib Javadi, Minnie Ho, Tom Mikkelsen

**Affiliations:** 1 Ontario Brain Institute; 2 Indoc Systems; 3 ICES

## Introduction

The Ontario Brain Institute (OBI) is a provincially funded, not-for-profit organization that accelerates discovery and innovation, benefiting both patients and the economy (Stuss, 2014). OBI has established a large-scale neuroinformatics platform - Brain-CODE - to support the collection, storage, federation, sharing, and analysis of different data types across several brain disorders (Behan et al., 2023; Vaccarino et al., 2018). A privacy preserving record linkage protocol was developed to allow for the linkage of research data at Brain-CODE with health administrative data holdings at the Institute for Clinical Evaluative Sciences (ICES) (Gee et al., 2018).

## Objective and Approach

The methodology related to an ongoing crosswalk linkage between OBI and ICES, to allow for more seamless integration between the respective data holdings, has been previously presented (Behan et al., 2020). This methodology has since been operationalized leading to the establishment of an ongoing crosswalk linkage that is updated on an annual basis.

## Results

Since the initial development of this ongoing crosswalk, two updates have been successfully completed leading to the linkage of over 7,000 study participants between the two platforms. This has led to a more efficient utilization of human and computational resources, compared to earlier data linkage projects completed on a project-by-project basis. Analysis projects in the areas of neurodegeneration, concussion, and neurodevelopmental disorders have already leveraged this crosswalk linkage process.

### Conclusions/Implications

The establishment of this ongoing crosswalk linkage has supported a more streamlined approach of data linkage activities between OBI and ICES allowing for enhanced neuroscience-focused research activities.

